# EEG Correlates of suicidal intentions in depressive patients who survived and have not been ill with COVID-19

**DOI:** 10.1192/j.eurpsy.2023.739

**Published:** 2023-07-19

**Authors:** A. F. Iznak, E. V. Iznak, E. V. Damyanovich

**Affiliations:** Laboratory of Neurophysiology, Mental Health Research Centre, Moscow, Russian Federation

## Abstract

**Introduction:**

The COVID-19 pandemic has caused an increase in suicidal intentions and attempts. One of the ways to prevent suicides is the timely detection of suicidal intentions. In this regard, it seems relevant to search for objective markers of suicidal intentions.

**Objectives:**

The aim of the study is to identify EEG correlates of suicidal intentions in depressive patients who survived and not have been ill with COVID-19.

**Methods:**

The study included 30 female patients aged 16-25 years with depressive disorders (F31.3-4, F21.3-4 + F34.0, according to ICD-10), who had previously a mild or asymptomatic coronavirus infection (group “COVID”), and 40 depressive patients, matched by sex, age, syndrome and the pre-treatment severity of depression (according to the total score of the Hamilton HDRS-17 scale), but not sick with COVID-19 (“non-COVID” group, The severity of suicidal intentions was quantified by the number of points of item 3 of the HDRS-17 scale. All patients underwent pre-treatment registration of the background EEG in a state of quiet wakefulness with eyes closed in 16 monopolar leads: F7, F3, F4, F8, T3, C3, Cz, C4, T4, T5, P3, Pz, P4, T6, O1 and O2. The analysis of the absolute EEG spectral power (SP) was carried out in 8 narrow frequency sub-bands. Statistical processing of the data obtained was carried out using the methods of descriptive statistics, comparison of means and correlation analysis of the IBM SPSS Statistics, v.22 software package.

**Results:**

In the “COVID” group, the mean number of points of item 3 of the HDRS-17 scale was slightly higher than in the “non-COVID” group (2.1 ± 1.5 and 1.8 ± 1.6, respectively), but these differences did not reach the level of statistical significance (p> 0.05). However, the structure of correlations between the values of this indicator of the severity of suicidal intentions and EEG parameters in the two groups turned out to be different. In the “COVID” group, the largest number of significantly (p<0.05) positive correlation coefficients was noted between the number of points of item 3 of the HDRS-17 scale and the SP values of slow-wave EEG components, e.g. theta2 sub-band (6-8 Hz) in leads F7, F3, F4, F8, T4, C3, C4, P3 and P4, as well as the delta sub-band (2-4 Hz) in leads F7 and P4. In the “non-COVID” group, the number of points of item 3 of the HDRS-17 scale positively correlated with the SP values of the alpha2 sub-band (9-11 Hz) in leads F7, F8, F4, C3, C4, T4, P4 and O2, as well as with SP values of the delta sub-band (2-4 Hz) in leads F7, F3 and C3.

**Conclusions:**

In depressive patients who underwent COVID-19, the severity of suicidal intentions is associated with EEG signs of a more reduced functional state of the cerebral cortex, including the anterior regions, than in patients who did not have coronavirus infection.

The study supported by the RSF grant No. 21-18-00129.

**Disclosure of Interest:**

None Declared

